# Supplementary material for: Retinoblastoma protein as an intrinsic BRD4 inhibitor modulates small molecule BET inhibitor sensitivity in cancer
Source: Nat Commun. 2022 Oct 23;13:6311. doi: 10.1038/s41467-022-34024-y (PMC9588789; doi:10.1038/s41467-022-34024-y)
Supplement: Supplementary file 1 — Supplementary Information [file 41467_2022_34024_MOESM1_ESM.pdf]

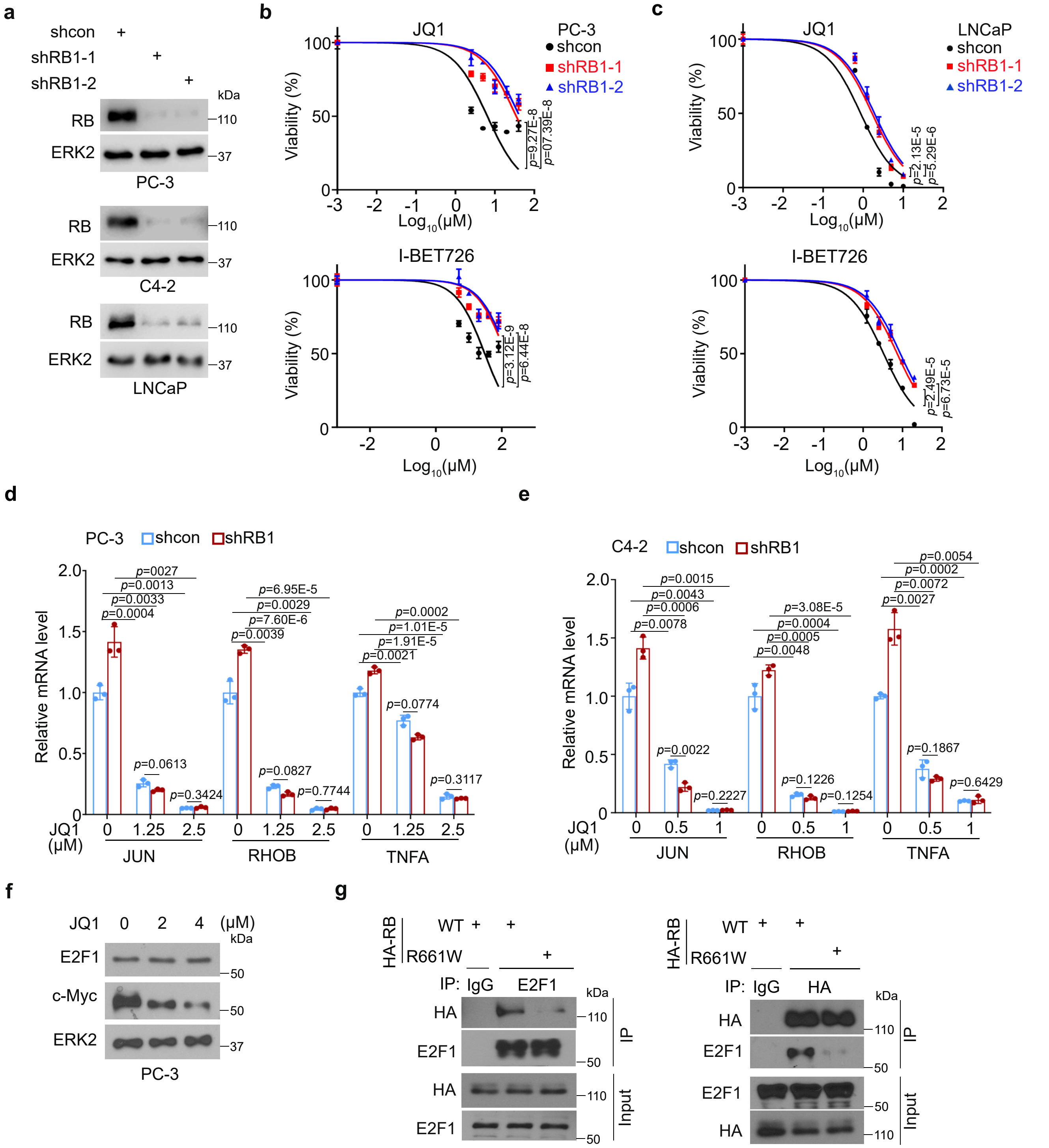

a
shcon
shRB1-1
shRB1-2
kDa
RB
ERK2
PC-3
C4-2
LNCaP
110
37
b
JQ1
I-BET726
PC-3
shcon
shRB1-1
shRB1-2
Viability (%)
Log10(μM)
p=9.27E-8
p=07.39E-8
p=3.12E-9
p=6.44E-8
c
LNCaP
p=2.13E-5
p=5.29E-6
p=2.49E-5
p=6.73E-5
d
PC-3
shcon
shRB1
Relative mRNA level
JQ1 (μM)
JUN
RHOB
TNFA
p=0027
p=0.0013
p=0.0033
p=0.0004
p=0.0613
p=0.3424
p=6.95E-5
p=0.0029
p=7.60E-6
p=0.0039
p=0.0827
p=0.7744
p=0.0002
p=1.01E-5
p=1.91E-5
p=0.0021
p=0.0774
p=0.3117
e
C4-2
p=0.0015
p=0.0043
p=0.0006
p=0.0078
p=0.0022
p=0.2227
p=3.08E-5
p=0.0004
p=0.0005
p=0.0048
p=0.1226
p=0.1254
p=0.0054
p=0.0002
p=0.0072
p=0.0027
p=0.1867
p=0.6429
f
JQ1
(μM)
E2F1
c-Myc
ERK2
PC-3
g
HA-RB
WT
R661W
IP:
IgG
E2F1
HA
IP
Input

Supplementary Fig. 1 The effects of RB loss, NF-κB pathway and E2F1 on PCa cell sensitivity to BET inhibitors. a, Western blot (WB) analysis of RB protein in PC-3, C4-2 and LNCaP cells infected with lentivirus for the indicated shRNAs for 48 h. ERK2, a load control for WB. **b, c,** Cell viability analysis in PC-3 (**b**) and LNCaP cells (**c**) infected with lentivirus for control or RB gene-specific shRNAs for 48 h followed by treatment of different doses of BET inhibitors for 72 h. **d, e,** RT-qPCR analysis of expression of NF-κB pathway genes in control and RB knockdown PC-3 (**d**) and C4-2 (**e**) cells treated with different doses of JQ1. **f,** WB analysis of indicated proteins in PC-3 cells treated with different doses of JQ1 for 36 h. **g,** WB analysis of indicated proteins in input and co-IP samples of IgG and anti-E2F1 antibody (left) or anti-HA antibody (right) from DU145 cells transfected with the indicated HA-tagged constructs for 24 h. Data in **b, c** represents mean ± s.d. from three independent experiments and statistical significance was determined using Two-way ANOVA. Data in **d**, **e** represent mean ± s.d. from three independent experiments and statistical significance was determined by two-sided Student's *t* test.

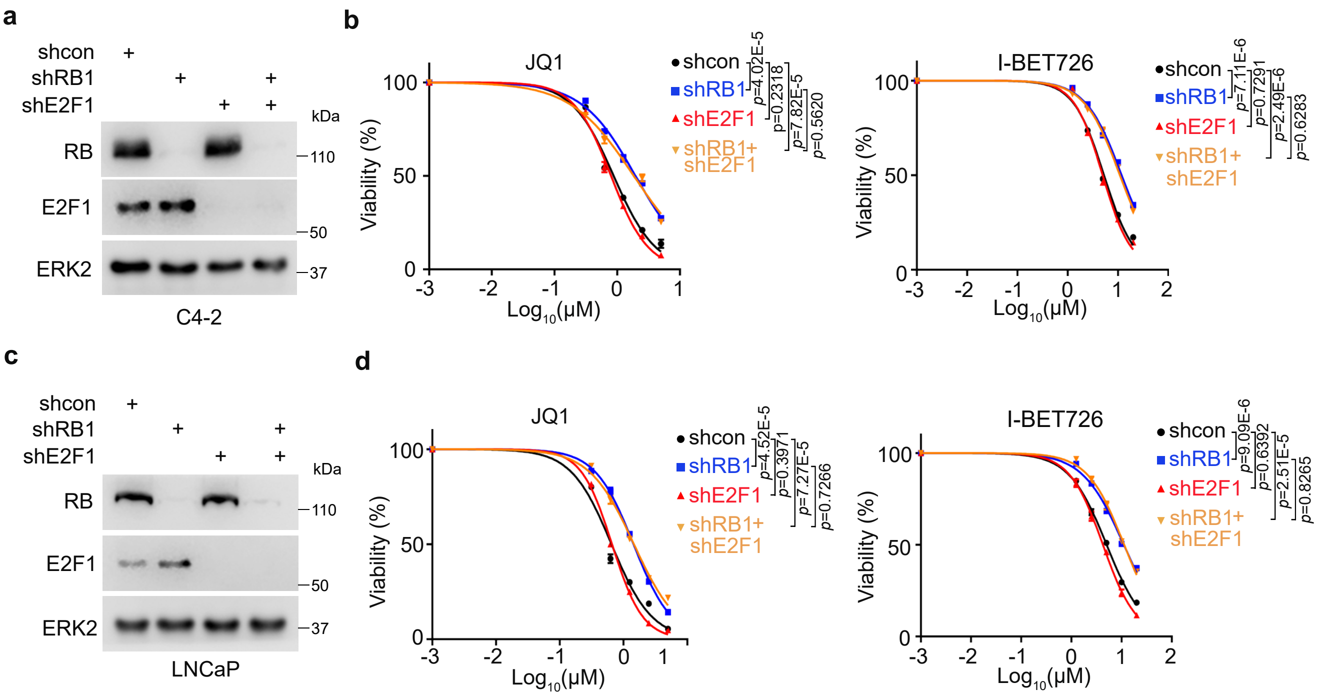

a
shcon
shRB1
shE2F1
kDa
RB
E2F1
ERK2
110
50
37
C4-2
b
JQ1
I-BET726
Viability (%)
Log10(μM)
shcon
shRB1
shE2F1
shRB1+ shE2F1
p=4.02E-5
p=0.2318
p=7.82E-5
p=0.5620
p=7.11E-6
p=0.7291
p=2.49E-6
p=0.6283
c
LNCaP
d
p=4.52E-5
p=0.3971
p=7.27E-5
p=0.7266
p=9.09E-6
p=0.6392
p=2.51E-5
p=0.8265

Supplementary Fig. 2 The effect of E2F1 on RB deficiency-induced BET inhibitor resistance in C4-2 or LNCaP cells. a-d, WB and cell viability analysis in C4-2 (**a, b**) and LNCaP cells (**c, d**) infected with lentivirus for control shRNA, RB shRNA and/or E2F1 shRNA for 48 h followed by treatment of different concentrations of BET inhibitors for 72 h. Data in **b** and **d** represent mean ± s.d. from three independent experiments and statistical significance was determined using Two-way ANOVA.

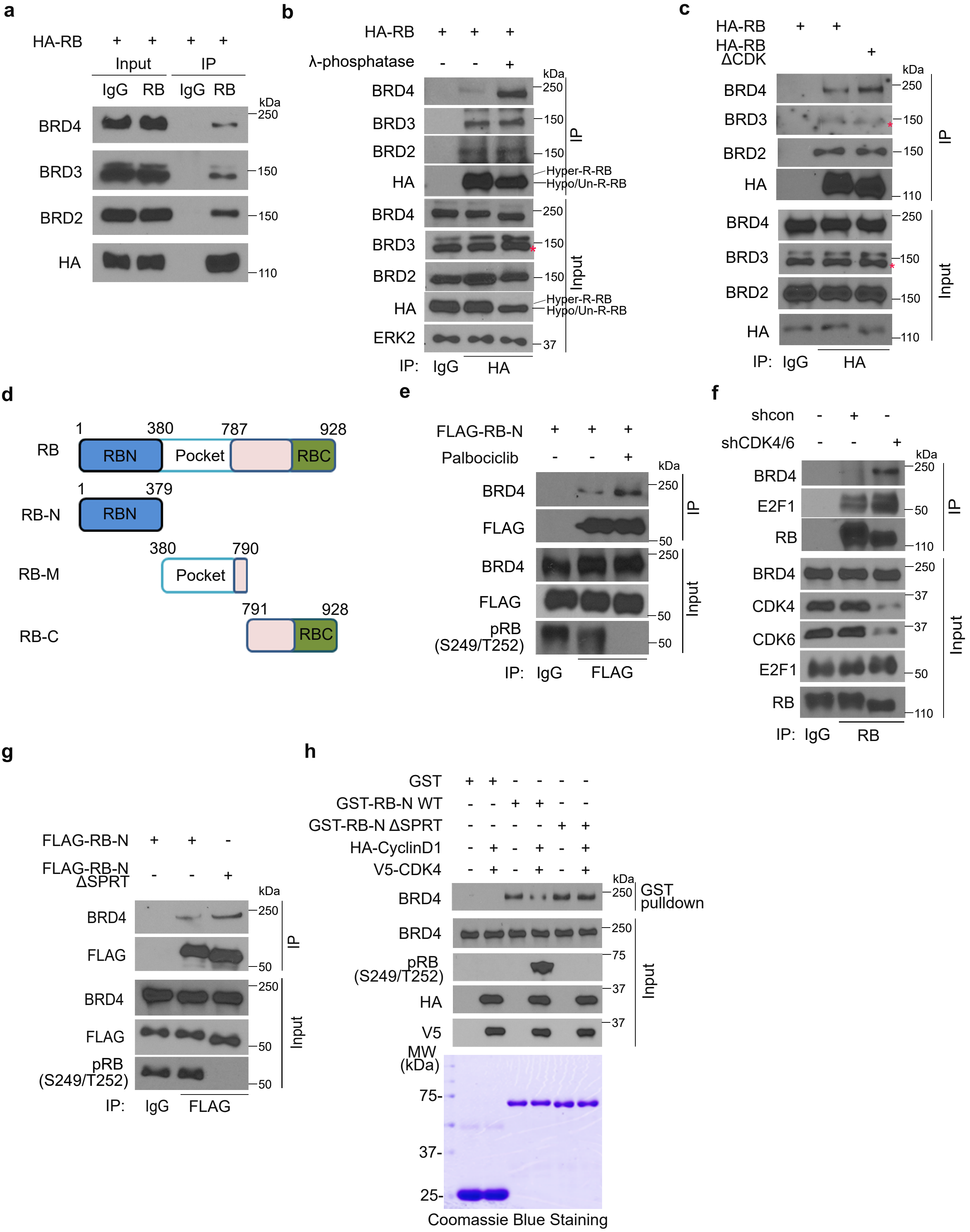

a
HA-RB + + + +
Input IP
IgG RB IgG RB
kDa
BRD4 250
BRD3 150
BRD2 150
HA 110
b
HA-RB + + +
λ-phosphatase - - +
kDa
BRD4 250
BRD3 150
BRD2 150
HA
Hyper-R-RB
Hypo/Un-R-RB
IP
BRD4 250
BRD3 150
BRD2 150
HA
Hyper-R-RB
Hypo/Un-R-RB
ERK2 37
Input
IP: IgG HA
c
HA-RB + +
HA-RB ΔCDK +
kDa
BRD4 250
BRD3 150
BRD2 150
HA 110
IP
BRD4 250
BRD3 150
BRD2 150
HA 110
Input
IP: IgG HA
d
RB
1 380 787 928
RBN Pocket RBC
RB-N
1 379
RBN
RB-M
380 790
Pocket
RB-C
791 928
RBC
e
FLAG-RB-N + + +
Palbociclib - - +
kDa
BRD4 250
FLAG 50
IP
BRD4 250
FLAG 50
pRB (S249/T252) 50
Input
IP: IgG FLAG
f
shcon - + -
shCDK4/6 - - +
kDa
BRD4 250
E2F1 50
RB 110
IP
BRD4 250
CDK4 37
CDK6 37
E2F1 50
RB 110
Input
IP: IgG RB
g
FLAG-RB-N + + -
FLAG-RB-N ΔSPRT - - +
kDa
BRD4 250
FLAG 50
IP
BRD4 250
FLAG 50
pRB (S249/T252) 50
Input
IP: IgG FLAG
h
GST + + - - - -
GST-RB-N WT - - + + - -
GST-RB-N ΔSPRT - - - - + +
HA-CyclinD1 - + - + - +
V5-CDK4 - + - + - +
kDa
BRD4 250
GST pulldown
BRD4 250
pRB (S249/T252) 75
HA 37
V5 37
Input
MW (kDa)
75-
37-
25-
Coomassie Blue Staining

Supplementary Fig. 3 RB dephosphorylation due to CDK4/6 inhibition enhances RB-BRD4 interaction. a, WB analysis of indicated proteins in input and co-IP samples of PC-3 cells transfected with the indicated HA-tagged RB. **b,** WB analysis of indicated proteins in input and co-IP samples of 293T cells transfected with the indicated HA-tagged RB. Cell lysate was treated with or without λ protein phosphatase prior to co-IP. **c,** WB analysis of indicated proteins in input and IP samples of 293T cells transfected with the indicated HA-tagged RB WT or phospho-resistant mutant RBΔCDK. **d,** Diagram showing GST recombinant protein constructs of three different RB fragments. **e,** WB analysis of indicated proteins in input and IP samples of 293T cells treated with or without palbociclib (5 μM) for 24 h. **f,** WB analysis of indicated proteins in input and IP samples of PC-3 cells infected with lentivirus for control or CDK4 and CDK6-specific shRNAs. **g,** WB analysis of indicated proteins in input and IP samples of PC-3 cells transfected with HA-tagged RB-N WT and RB-NΔSPRT mutant. **h,** WB analysis of indicated proteins in input and GST pulldown samples. GST or GST-RB-N and mutant recombinant proteins purified from bacteria were first subjected to in vitro kinase assay using V5-CDK4/6 and HA-Cyclin D immunoprecipitated from PC-3 cells after transfection with corresponding constructs. Western blot assays in **a, b, c, e** and **f** were repeated two times independently with similar results.

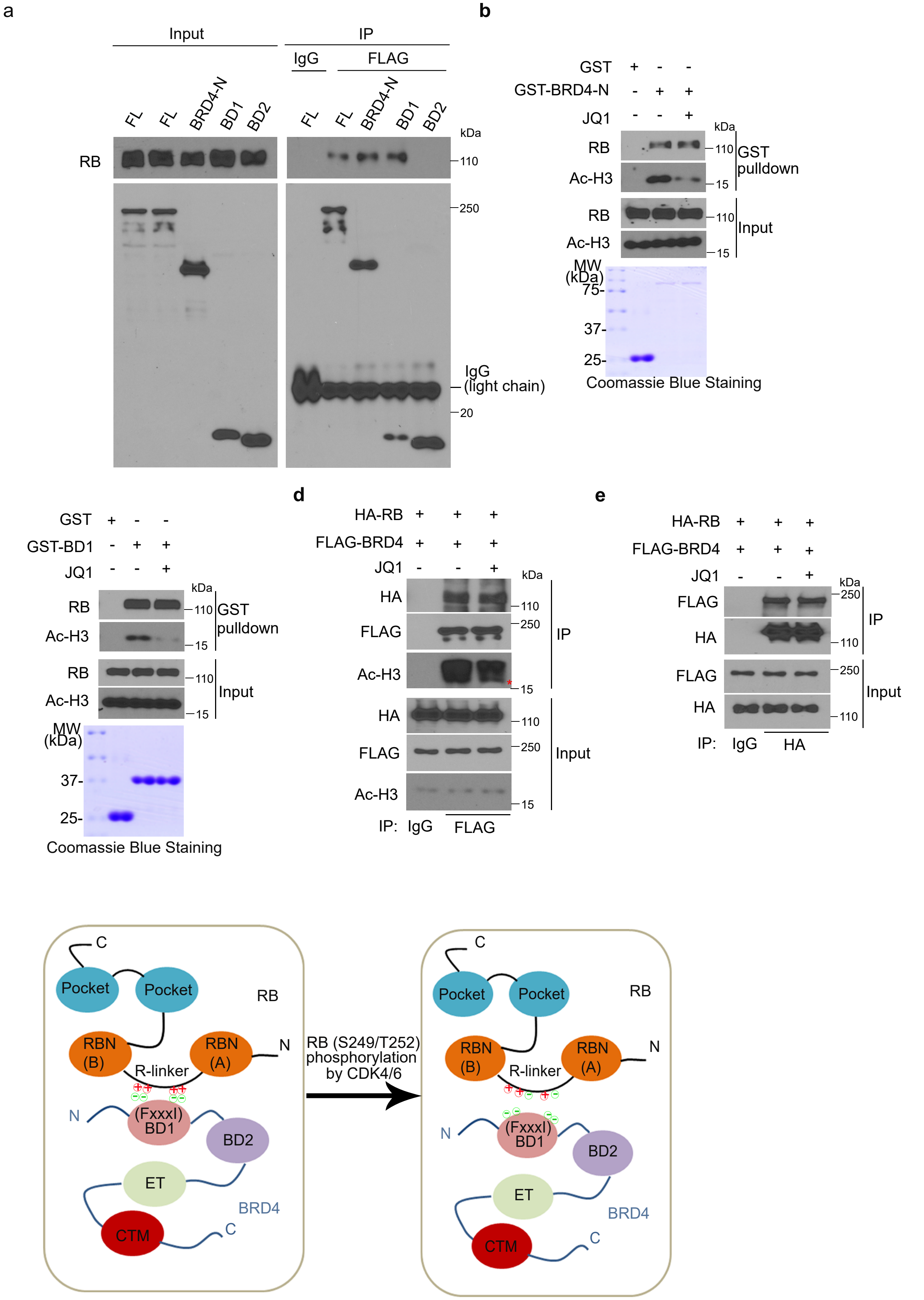
a
Input
IP
IgG
FLAG
FL
FL
BRD4-N
BD1
BD2
FL
FL
BRD4-N
BD1
BD2
kDa
RB
110
250
IgG
(light chain)
20
b
GST + - -
GST-BRD4-N - + +
JQ1 - - +
kDa
RB
Ac-H3
110
15
GST
pulldown
RB
Ac-H3
110
15
Input
MW
(kDa)
75-
37-
25-
Coomassie Blue Staining
c
GST + - -
GST-BD1 - + +
JQ1 - - +
kDa
RB
Ac-H3
110
15
GST
pulldown
RB
Ac-H3
110
15
Input
MW
(kDa)
37-
25-
Coomassie Blue Staining
d
HA-RB + + +
FLAG-BRD4 + + +
JQ1 - - +
kDa
HA
FLAG
Ac-H3
110
250
15
IP
HA
FLAG
Ac-H3
110
250
15
Input
IP: IgG FLAG
e
HA-RB + + +
FLAG-BRD4 + + +
JQ1 - - +
kDa
FLAG
HA
250
110
IP
FLAG
HA
250
110
Input
IP: IgG HA
f
C
Pocket
Pocket
RB
RBN
(B)
RBN
(A)
N
R-linker
N
(Fxxxl)
BD1
BD2
ET
CTM
BRD4
C
RB (S249/T252)
phosphorylation
by CDK4/6
C
Pocket
Pocket
RB
RBN
(B)
RBN
(A)
N
R-linker
N
(Fxxxl)
BD1
BD2
ET
CTM
BRD4
C

Supplementary Fig. 4 RB interacts with BD1 and the interaction is not influenced by JQ1. **a,** WB analysis of indicated proteins in input and IP samples of PC-3 cells transfected with indicated FLAG-tagged full-length BRD4 or mutants. **b,** WB analysis of indicated proteins in input and samples pulled down by GST or GST-BRD4-N recombinant proteins purified from bacteria from lysate of PC-3 cells. 1 μM JQ1 was added into the lysate during the incubation. **c,** WB analysis of indicated proteins in input and samples pulled down by GST or GST-BD1 recombinant proteins purified from bacteria from lysate of PC-3 cells. 1 μM JQ1 was added into the lysate during the incubation. **d, e,** WB analysis of indicated proteins in input and IP samples of PC-3 cells transfected with HA-RB and FLAG-BRD4. 1 μM JQ1 was added into the lysate prior to the co-IP assay. Anti-FLAG (**d**) and anti-HA (**e**) antibodies were used for IP. Data in **a-e** were repeated two times independently with similar results. **f,** A hypothetical model depicting a notion that the negatively charged amino acids surrounding the FXXXI motif are the determinants for the interaction between RB and BRD4 and that such interaction is abolished by CDK4/6-mediated S249/T252 phosphorylation in the R-linker region of the RB.

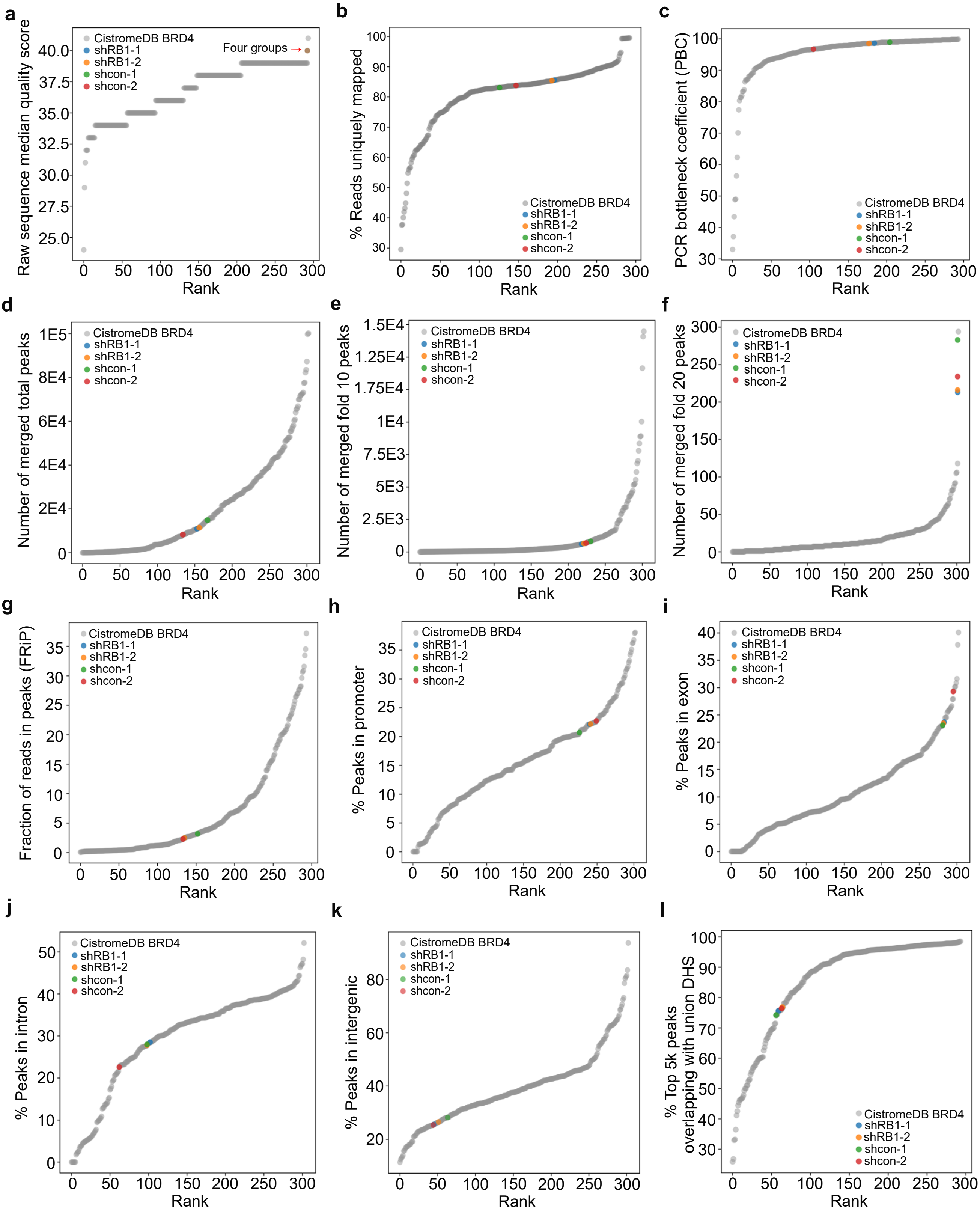

a
Raw sequence median quality score
CistromeDB BRD4
shRB1-1
shRB1-2
shcon-1
shcon-2
Four groups
Rank
b
% Reads uniquely mapped
Rank
c
PCR bottleneck coefficient (PBC)
Rank
d
Number of merged total peaks
Rank
e
Number of merged fold 10 peaks
Rank
f
Number of merged fold 20 peaks
Rank
g
Fraction of reads in peaks (FRiP)
Rank
h
% Peaks in promoter
Rank
i
% Peaks in exon
Rank
j
% Peaks in intron
Rank
k
% Peaks in intergenic
Rank
l
% Top 5k peaks overlapping with union DHS
Rank

Supplementary Fig. 5 Plots of BRD4 ChIP-seq metrics determined by analysis using Cistrome pipeline. Comparison of raw sequence median quality score (**a**), % Reads uniquely mapped (**b**); PCR bottleneck coefficient (PBC) (**c**), Number of merged Total (**d**) /Fold 10 (**e**) /Fold 20 peaks (**f**), Fraction of reads in peaks (FRiP) (**g**), % Peaks in promoter (**h**) /exon (**i**) /intron (**j**) /intergenic (**k**), and % Top 5k peaks overlapping with union DHS (**l**) of the BRD4 ChIP-seq data in C4-2 cells expressing control shRNAs (shcon) or RB-specific shRNAs (shRB) to the BRD4 ChIP-seq results of 294 different human cell samples in the Cistrome database (DB).

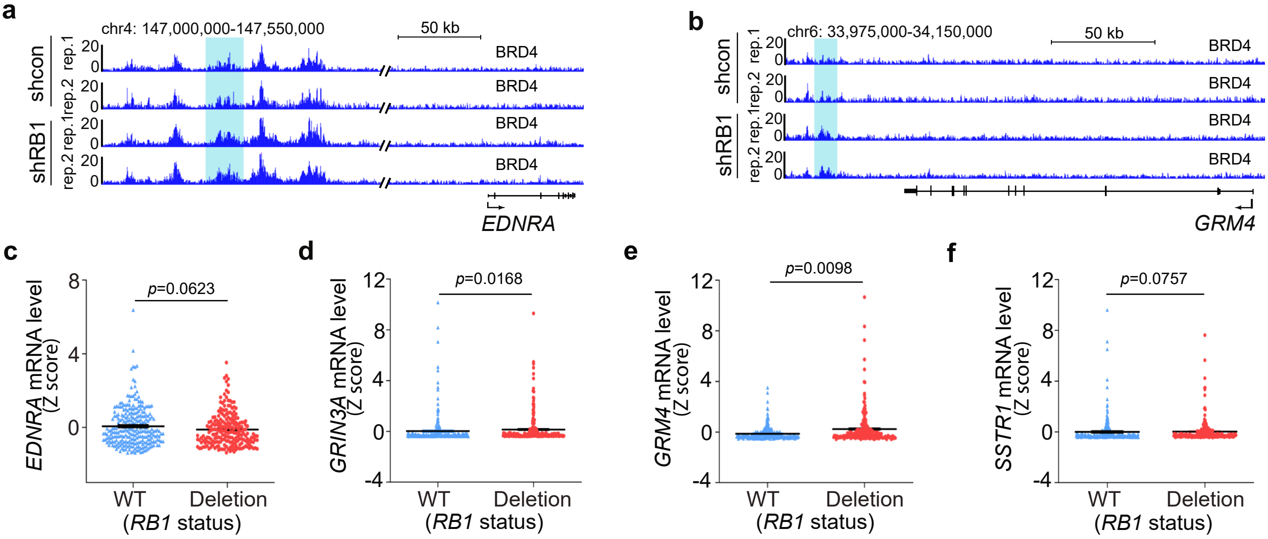
a
chr4: 147,000,000-147,550,000
50 kb
shcon
rep.1
rep.2
shRB1
BRD4
EDNRA
b
chr6: 33,975,000-34,150,000
50 kb
GRM4
c
EDNRA mRNA level (Z score)
p=0.0623
WT
Deletion
(RB1 status)
d
GRIN3A mRNA level (Z score)
p=0.0168
e
GRM4 mRNA level (Z score)
p=0.0098
f
SSTR1 mRNA level (Z score)
p=0.0757

Supplementary Fig. 6 RB regulation of GPCR-cAMP pathway genes. a, b, UCSC Genome Browser screenshots showing the increased BRD4 occupancy in a region in the *EDNRA* (**a**) *and GRM4* (**b**) gene loci in cells expressing shRB compared to shcon cells. **c-f,** Meta-analysis of RNA-seq data showing the association of increased expression of *EDNRA* (**c**), *GRIN3A* (**d**), *GRM4* (**e**), and *SSTR1* (**f**) with the different genetic statuses of *RB1* gene in PCa samples of the TCGA cohort. N=267 for the RB 'WT' group and n=218 for the RB 'Deletion' group in **c-f**. Data was shown as mean ± s.d. in **c-f**. in *p* values for data in **c-f** were determined using two-tailed Mann-Whitney U test.

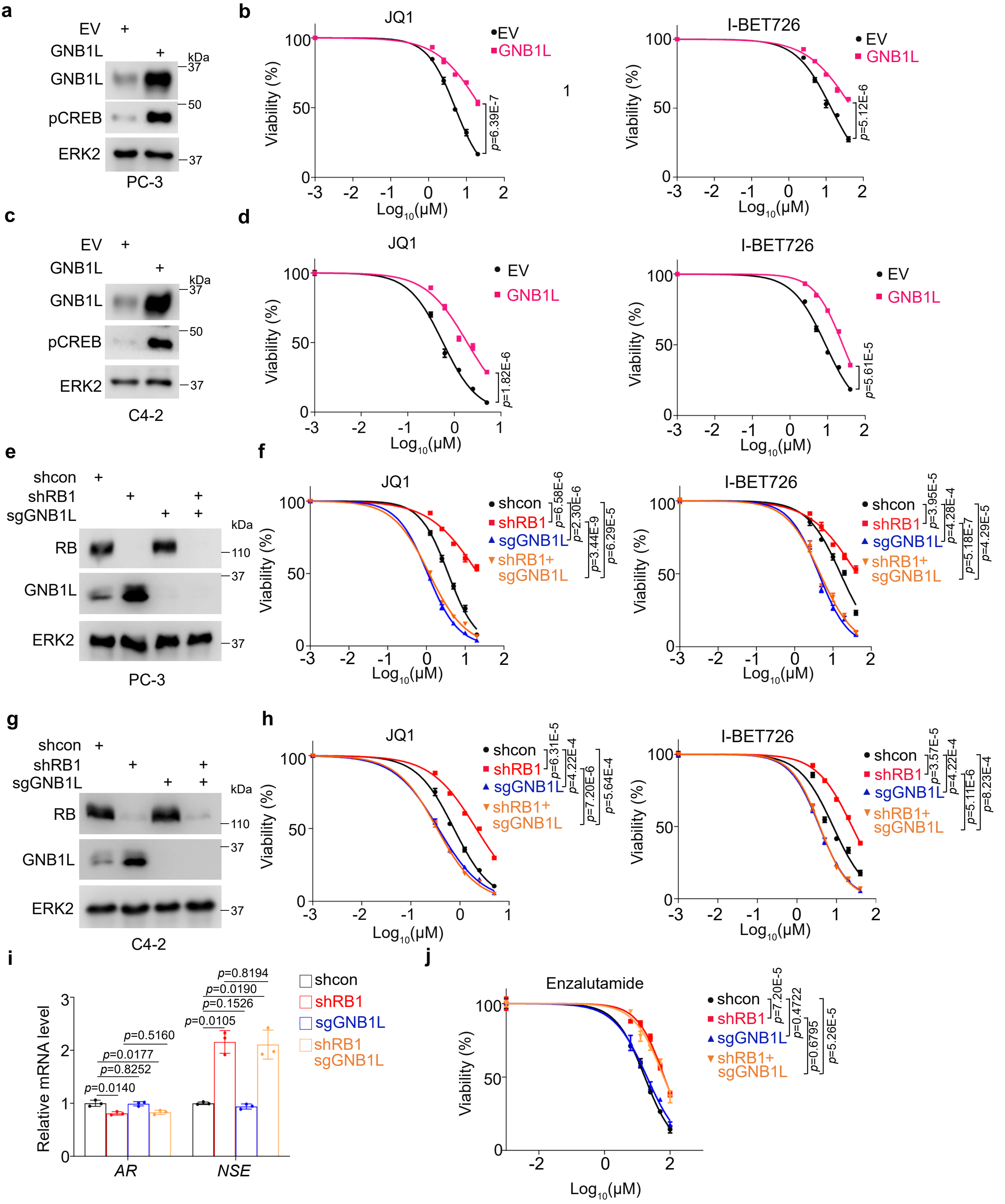
a
EV
GNB1L
GNB1L
pCREB
ERK2
kDa
37
50
37
PC-3
b
JQ1
EV
GNB1L
Viability (%)
Log10(μM)
p=6.39E-7
1
I-BET726
p=5.12E-6
c
C4-2
d
p=1.82E-6
p=5.61E-5
e
shcon
shRB1
sgGNB1L
RB
110
f
shRB1+
p=6.58E-6
p=2.30E-6
p=3.44E-9
p=6.29E-5
p=3.95E-5
p=4.28E-4
p=5.18E-7
p=4.29E-5
g
h
p=6.31E-5
p=4.22E-4
p=7.20E-6
p=5.64E-4
p=3.57E-5
p=5.11E-6
p=8.23E-4
i
Relative mRNA level
AR
NSE
p=0.0140
p=0.8252
p=0.0177
p=0.5160
p=0.0105
p=0.1526
p=0.0190
p=0.8194
j
Enzalutamide
p=7.20E-5
p=0.4722
p=0.6795
p=5.26E-5

Supplementary Fig. 7 GNB1L regulates BET inhibitor resistance in PCa cells. a, WB analysis of indicated proteins in PC-3 cells transfected with plasmids for empty vector (EV) or GNB1L for 24 h. **b,** Cell viability analysis in PC-3 cells transfected with indicated plasmids for 24 h followed by treatment with different concentrations of BET inhibitors JQ1 and I-BET726 for 72 h. **c,** WB analysis of indicated proteins in C4-2 cells transfected with plasmids for empty vector (EV) or GNB1L for 24 h. **d,** Cell viability analysis in C4-2 cells transfected with indicated plasmids for 24 h followed by treatment with different concentrations of BET inhibitors JQ1 and I-BET726 for 72 h. **e-h,** WB and cell viability analysis in PC-3 (**e, f**) and C4-2 cells (**g, h**) infected with lentivirus for control or RB shRNAs or sgRNAs for GNB1L for 48 h followed by treatment with different doses of BET inhibitors for 72 h. **i**, RT-qPCR analysis of *AR* and *NSE* mRNA expression in C4-2 cells infected lentivirus as in (**g**) for 48 h. **j**, Cell viability analysis in C4-2 cells infected with lentivirus as in (**g**) followed by treatment with different concentrations of enzalutamide. Data in **i** was shown as mean ± s.d. from three independent experiments. Data in **b**, **d**, **f** and **h** was shown as mean ± s.d. from five replicates. Data in **j** was shown as mean ± s.d. from three replicates. Statistical significance in **b**, **d**, **f, h** and **j** is calculated by Two-ANOVA analysis. Statistical significance in **i** is calculated by Two-sided Student's *t* test.

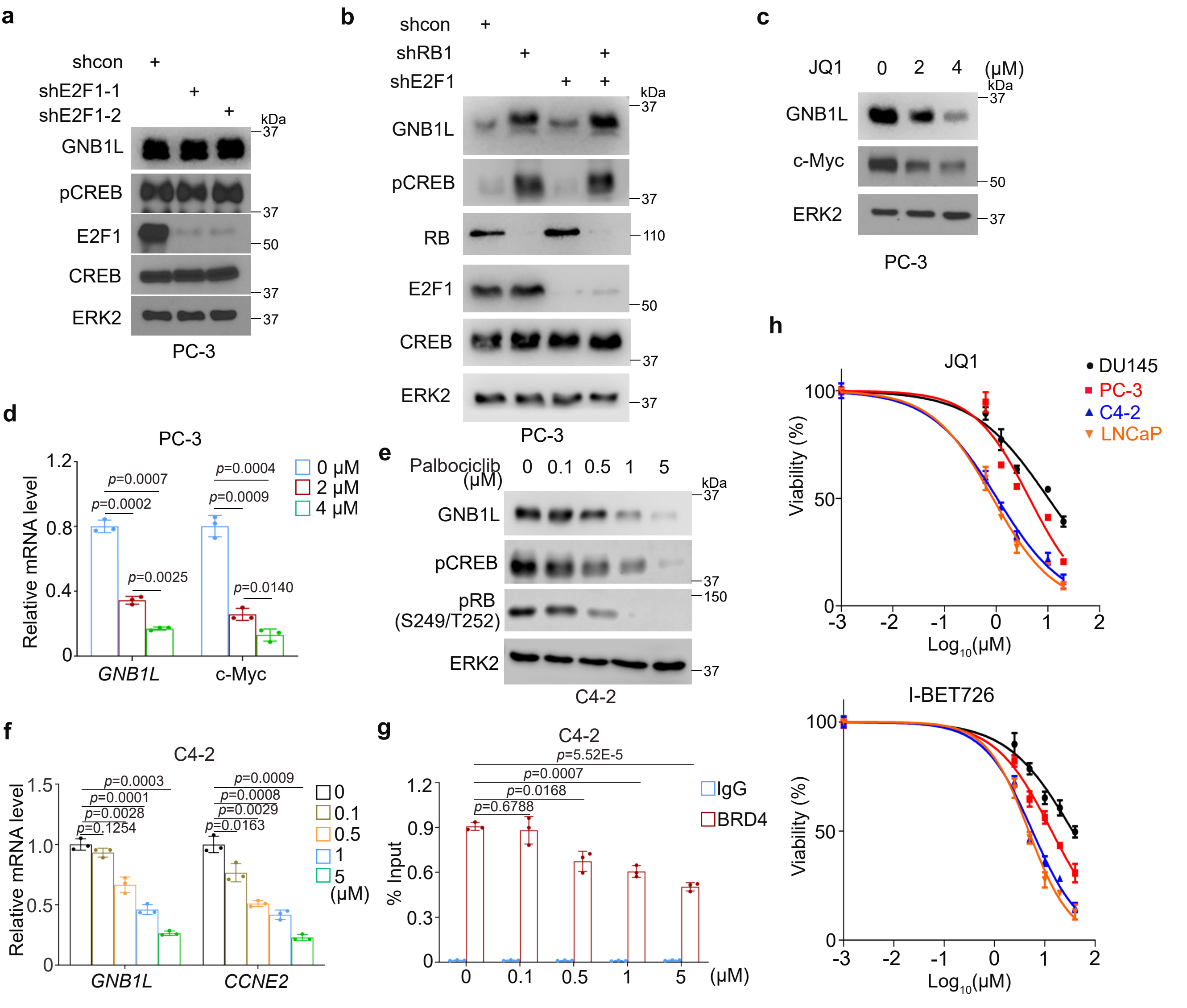

i

	JQ1 IC_{50} (μM)	I-BET726 IC_{50} (μM)
DU145	10.21	35.97
PC-3	4.41	13.71
C4-2	1.04	5.67
LNCaP	0.86	4.80

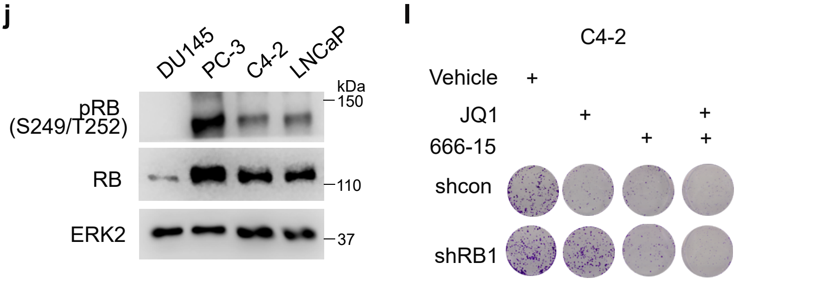

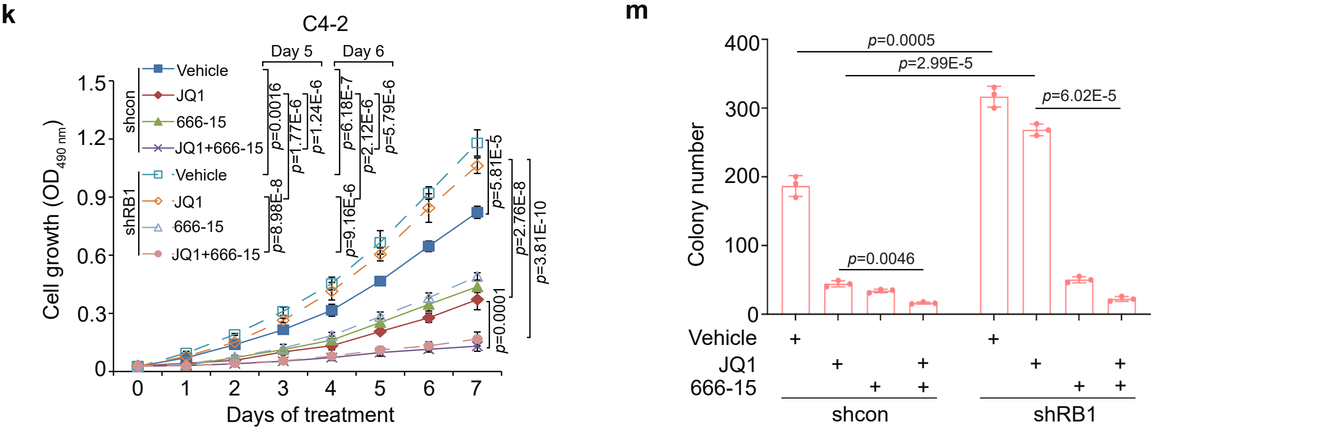

Supplementary Fig. 8 Regulation of GNB1L expression by JQ1, the effects of RB status on BET inhibitor sensitivity in PCa cell lines and the effect of CREB inhibitor on JQ1 sensitivity in RB-deficient PCa cells. a, b, WB analysis of indicated proteins in PC-3 cells infected with lentivirus for the indicated shRNAs for 48 h. **c, d,** WB (**c**) and RT-qPCR analysis (**d**) of GNB1L expression in PC-3 cells treated with different doses of JQ1 for 36 h. **e, f** WB (**e**) and RT-qPCR (**f**) analysis of GNB1L protein and mRNA expression in C4-2 cells treated with vehicle or different doses of palbociclib for 36 h. **g,** ChIP-qPCR analysis of BRD4 occupancy in the *GNB1L* gene locus in C4-2 cells treated with vehicle or different doses of palbociclib as in (**e**,**f**). **h-j,** Cell viability (**h**), IC50 (**i**) and WB analysis (**j**) of indicated proteins in PCa cell lines DU145, PC-3, C4-2 and LNCaP treated with different doses of BET inhibitors JQ1 (Top) and I-BET726 (Bottom) for 72 h in **h**. **k,** Cell growth assay in control or RB knockdown C4-2 cells treated with JQ1 (0.5 µM) or 666-15 (0.5 µM) alone or in combination for the indicated time periods. Statistical analyses were performed at Day 5, 6 and 7 time points. **l, m,** Colony formation assay in control or RB knockdown C4-2 cells in the presence of indicated inhibitors for 14 days and colonies were photographed (**l**) and quantified (**m**). Western blot assays in **a-c, e and j** were repeated two times independently with similar results. Data in **d**, **f**, **g**, **h** and **m** are shown as mean ± s.d. from three independent experiments. Data in **k** is shown as mean ± s.d. from five replicates. Statistical significance in **d**, **f**, **g**, **k** and **m** was determined using two-sided Student's *t* test.

Supplementary Data 1. BRD4 ChIP-seq quality metrics determined by Cistrome analysis

Samples	Raw sequence median quality score	Total Reads	Mapped_rate	% Reads uniquely mapped	PBC	Number of merged Total peaks	Number of merged Fold 10 peaks	Number of merged Fold 20 peaks	FRiP	% Peaks in promoter	% Peaks in exon	% Peaks in intron	% Peaks in intergenic	% Top 5k peaks overlapping with union DHS
shcon-1	40	84726877	98.97%	83.08%	0.9893	14880	815	283	3.2	0.206823465	0.231232053	0.279912485	0.282031998	0.7424
shcon-2	40	77495615	99.44%	83.77%	0.967	8239	685	234	2.3	0.226979399	0.292876644	0.225986597	0.254157359	0.7644
shRB1-1	40	89100117	99.25%	85.55%	0.9861	10937	600	213	2.5	0.221433243	0.237036345	0.285153695	0.256376717	0.7564
shRB1-2	40	88591649	99.28%	85.40%	0.9853	11400	651	216	2.5	0.221864376	0.235015208	0.278135624	0.264984792	0.7684

Supplementary Data 2. The genome loci with increased BRD4 occupancy after RB knockdown.

Index	ID	log2/ FoldChange	padj	Target_gene
2	chr1:20249800:20254921	2.385	0.0000	VWA5B1(-38558),UBXN10(+66276)
3	chr18:42641279:42647679	1.438	0.0000	RIT2(+471212),PIK3C3(+689273)
4	chr18:58786029:58791952	1.454	0.0000	ZNF532(-73609),MALT1(+117605)
5	chr18:73529674:73535636	1.317	0.0000	NETO1(-664706),FBXO15(+615210)
6	chr18:73658755:73663509	1.773	0.0000	NETO1(-793183),FBXO15(+486733)
7	chr1:20255284:20259457	2.252	0.0000	VWA5B1(-33548),UBXN10(+71286)
9	chr18:42851520:42855412	2.010	0.0000	RIT2(+262225),PIK3C3(+898260)
10	chr21:28695414:28702152	1.119	0.0000	N6AMT1(+186588)
11	chr3:177633438:177637371	1.617	0.0000	KCNMB2(-923295),TBL1XR1(-438927)
12	chr18:73503077:73505505	1.646	0.0000	NETO1(-636342),FBXO15(+643574)
13	chr7:25384245:25389712	1.037	0.0000	NFE2L3(-765261),NPVF(-158493)
18	chr3:174346626:174350128	1.353	0.0000	NAALADL2(-510903),NLGN1(+949929)
20	chr7:90485071:90490380	1.156	0.0000	CDK14(-108771),CLDN12(+84340)
25	chr18:43138800:43143293	1.045	0.0000	RIT2(-25356),SYT4(+136603)
44	chr11:23287151:23288919	1.555	0.0000	SVIP(-458170)
50	chr18:42232793:42236133	1.023	0.0000	PIK3C3(+279257),RIT2(+881228)
53	chr1:20475710:20477952	2.065	0.0000	CAMK2N1(+9389),VWA5B1(+185912)
57	chr8:133899005:133901764	1.643	0.0000	ST3GAL1(-328460),ZFAT(+812653)
61	chr4:147084573:147089818	1.013	0.0000	EDNRA(-393736),POU4F2(+448303)
66	chr12:102490040:102493037	1.307	0.0000	IGF1(-10939),PAH(+426064)
72	chr3:167898769:167901852	1.016	0.0000	SERPINI1(+164588),GOLIM4(+195664)
73	chr2:179446092:179448200	1.577	0.0000	SESTD1(-182356),ZNF385B(+414359)
81	chr18:73625417:73627506	1.025	0.0000	NETO1(-758513),FBXO15(+521403)
88	chr12:91952841:91955533	1.013	0.0001	DCN(-771064),BTG1(+191710)
91	chr1:221209356:221211588	1.295	0.0001	HLX(+331072),DUSP10(+531704)
95	chr1:20479886:20482651	1.854	0.0001	CAMK2N1(+4951),VWA5B1(+190350)
100	chr9:114979587:114982232	1.105	0.0001	TNFSF8(-50493),TNC(+137347)
116	chr14:29964208:29965664	1.296	0.0003	G2E3(-594187),PRKD1(-37294)
118	chrX:121120179:121123320	1.152	0.0003	GLUD2(+74162)
119	chr1:221249114:221250787	1.809	0.0003	HLX(+370551),DUSP10(+492225)
123	chr5:36441753:36443118	1.391	0.0003	SLC1A3(-163919),RANBP3L(-140536)
124	chr12:91383850:91385156	1.534	0.0004	DCN(-201380),BTG1(+761394)
131	chr10:110857271:110859937	1.168	0.0005	PDCD4(-13203),RBM20(+214207)
133	chr4:147123172:147125089	1.606	0.0005	EDNRA(-356801),POU4F2(+485238)
151	chr11:23391105:23392068	1.847	0.0008	SVIP(-561722)
155	chr18:43562907:43564629	1.180	0.0011	SYT4(-286118)
158	chr9:114995407:114997603	1.230	0.0012	TNFSF8(-66088),TNC(+121752)
160	chr18:73317679:73318541	1.870	0.0014	NETO1(-450161),FBXO15(+829755)
170	chr22:19934627:19937027	1.261	0.0015	GNB1L(-80888),COMT(-5780)
171	chr11:86933962:86936592	1.114	0.0016	FZD4(+20114),PRSS23(+135037)
173	chr4:100584302:100585774	1.469	0.0017	EMCN(-66945),PPP3CA(+762442)

177	chr18:43174371:43175606	1.336	0.0019	RIT2(-59298),SYT4(+102661)
184	chr3:177419606:177421697	1.164	0.0023	TBL1XR1(-224174)
186	chr6:41766314:41769129	1.121	0.0025	PGC(-20325),FRS3(+12178)
193	chr4:147127327:147128682	1.473	0.0028	EDNRA(-352927),POU4F2(+489112)
197	chr18:42855477:42856723	1.876	0.0029	RIT2(+259591),PIK3C3(+900894)
201	chr14:38868066:38869654	1.182	0.0035	CLEC14A(-612491),SEC23A(+234668)
203	chr6:110528643:110529530	1.371	0.0035	SLC22A16(-52446),CDK19(+286229)
206	chr7:90460204:90461711	1.514	0.0042	CDK14(-135539),CLDN12(+57572)
213	chr18:59012573:59013951	1.374	0.0044	SEC11C(-126215),ZNF532(+150662)
214	chr5:37088248:37091474	1.075	0.0044	C5orf42(+159567),NIPBL(+213102)
219	chr3:178134607:178136025	1.747	0.0046	TBL1XR1(-938838),KCNMB2(-423384)
218	chr1:221246735:221248971	1.047	0.0046	HLX(+368453),DUSP10(+494323)
222	chr1:221244392:221246613	1.087	0.0048	HLX(+366103),DUSP10(+496673)
240	chr1:105397653:105399432	1.129	0.0064	NONE
241	chr8:87169048:87171027	1.054	0.0066	CNGB3(-426363)
244	chr3:167935866:167937937	1.072	0.0069	GOLIM4(+159073),SERPINI1(+201179)
250	chr4:100421271:100422325	1.355	0.0081	DDIT4L(-231330),EMCN(+96295)
258	chr3:174948885:174950497	1.161	0.0097	NAALADL2(+90411)
263	chr6:138477322:138479587	1.087	0.0105	HEBP2(+74236),NHSL1(+94085)
266	chr1:20212256:20213522	1.887	0.0110	VWA5B1(-78030),UBXN10(+26804)
268	chr12:91389457:91390399	1.595	0.0110	DCN(-206805),BTG1(+755969)
269	chr4:100510531:100511580	1.713	0.0110	DDIT4L(-320588),EMCN(+7037)
271	chr4:101888230:101889009	1.500	0.0111	BANK1(+98013),SLC39A8(+456519)
276	chr6:33987561:33989573	1.181	0.0125	MLN(-184556),GRM4(+157513)
281	chr4:100433095:100435823	1.148	0.0128	DDIT4L(-243991),EMCN(+83634)
287	chr1:222694228:222695708	1.145	0.0139	AIDA(+17556),MIA3(+76882)
295	chr8:74734582:74735785	1.539	0.0157	PI15(-89353),GDAP1(+384802)
297	chr18:42052729:42054054	1.337	0.0160	PIK3C3(+98186)
309	chr18:42382980:42384241	1.280	0.0185	PIK3C3(+428405),RIT2(+732080)
321	chr21:28690808:28692316	1.222	0.0203	N6AMT1(+193809)
324	chr17:30039485:30041154	1.139	0.0205	NSRP1(-76461),EFCAB5(+98715)
329	chr15:57690597:57691819	1.519	0.0213	POLR2M(-15421),GCOM1(+99194)
331	chr3:175126678:175127563	1.285	0.0216	NAALADL2(+267841)
334	chr1:72000648:72001352	1.489	0.0218	ZRANB2(-919703),NEGR1(+281734)
342	chr6:39089134:39091660	1.004	0.0222	SAYSD1(+24792),GLP1R(+41599)
352	chr5:87731830:87732286	2.345	0.0248	CCNH(-319039),TMEM161B(+536772)
355	chr4:154837398:154839635	1.336	0.0254	NPY2R(-370112),RBM46(+57304)
357	chr12:91382021:91382707	1.295	0.0254	DCN(-199241),BTG1(+763533)
365	chr18:73169511:73170182	1.920	0.0266	NETO1(-301898),FBXO15(+978018)
368	chr18:73295735:73296234	2.099	0.0270	NETO1(-428036),FBXO15(+851880)
370	chr7:90490482:90490910	2.351	0.0272	CDK14(-105801),CLDN12(+87310)
371	chr1:47031179:47031989	1.270	0.0278	CYP4Z1(-35904),CYP4X1(+8016)
372	chr7:152988839:152990715	1.026	0.0278	ACTR3B(+230028)
387	chr6:49422440:49423401	1.787	0.0310	MUT(+40270)
388	chr16:53731676:53733489	1.211	0.0312	FTO(+28620),IRX3(+554180)
389	chr1:47047109:47047359	2.983	0.0316	CYP4Z1(-20254),CYP4X1(+23666)
400	chr8:8361862:8362993	1.251	0.0337	USP17L3(-384443),PRAG1(+24070)

399	chr4:58687818:58689089	1.180	0.0337	NONE
408	chr5:168600429:168601117	2.232	0.0356	PANK3(-21173),SLIT3(+700009)
407	chr3:103699298:103701063	1.043	0.0356	NONE
411	chr18:73168187:73168832	1.476	0.0365	NETO1(-300561),FBXO15(+979355)
412	chr1:119389998:119391608	1.089	0.0377	HSD3B2(-24128),HAO2(+21994)
414	chr3:177637596:177638718	1.623	0.0379	KCNMB2(-920543),TBL1XR1(-441679)
417	chr21:21343289:21343961	2.212	0.0390	NCAM2(+345310)
420	chr18:42626223:42627039	1.447	0.0393	RIT2(+489060),PIK3C3(+671425)
419	chr14:38866348:38867947	1.108	0.0393	CLEC14A(-610779),SEC23A(+236380)
424	chr4:100586083:100586677	1.842	0.0408	EMCN(-68287),PPP3CA(+761100)
428	chr3:176741032:176742187	1.242	0.0422	TBL1XR1(+454868)
427	chr1:119387855:119389060	1.054	0.0422	HSD3B2(-26473),HAO2(+19649)
433	chr1:222680865:222682494	1.238	0.0426	AIDA(+30844),MIA3(+63594)
446	chr5:165027387:165028714	1.047	0.0440	NONE
447	chr8:20083660:20084876	1.229	0.0449	SLC18A1(+98938),LPL(+145297)
452	chrX:121123819:121125004	1.667	0.0475	GLUD2(+76824)
455	chr15:92644338:92646321	1.024	0.0478	FAM174B(+10628),ST8SIA2(+251502)
458	chr14:28935659:28936557	1.295	0.0492	FOXG1(+170720),PRKD1(+991534)
459	chr2:179443011:179444578	1.520	0.0493	SESTD1(-179005),ZNF385B(+417710)

Note: Data was collected for differential analysis by DEseq2, calculated for p value by two-sided Wald test after modeling the count data by logistic regression, and p value was adjusted by the benjamini and Hochberg method for multiple comparison

Supplementary Data 3. The genome loci with down-regulated BRD4 occupancy after RB knockdown.

Index	ID	log2FoldCl	padj	Target_gene
220	chr18:2026	-1.271032	0.004667	ROCK1(+844959)
333	chr11:5224	-1.584986	0.02167	OR51V1(-24566),HBB(+1805)
339	chr2:92801	-1.103303	0.022063	NONE
364	chr11:5151	-1.169591	0.026598	NONE

Note: Data was collected for differential analysis by DEseq2, calculated for p value by two-sided Wald test after modeling the count data by logistic regression, and p value was adjusted by the benjamini and Hochberg method for multiple comparison

Supplementary Data 4. Sequence information of PCR primers, shRNAs and sgRNAs.

**RT-qPCR primers.**		
Gene name	Forward (F) /Reverse (F	Sequence (5'-3')
GNB1L	F	GTTCCCGACTCCGGCAAG
GNB1L	R	GGATGCAGTTACCTGAGGCT
EDNRA	F	ACATCTTAAGCAGCGTCGAGA
EDNRA	R	TCGGTTCTTGTCCATCTCGTT
SSTR1	F	CAACTCTCCAGGCTTAGGGC
SSTR1	R	AGTGCATGTGCGGTCTGTTA
GRIN3A	F	CCACACCAGCCAGAGATTACA
GRIN3A	R	CATACGGTAAGCTGACGGGG
GRM4	F	CCTTTGGATTTTGCCGGTGT
GRM4	R	GTAGCTGATCTGGGGTATCTGA
RB1	F	TTTCTGCTTTTGCATTCGTG
RB1	R	GGAAGCAACCCTCCTAAACC
BRD4	F	AGCAGCAACAGCAATGTGAG
BRD4	R	GCTTGCACTTGTCCTCTTCC
c-Myc	F	TACAACACCCGAGCAAGGAC
c-Myc	R	AGGCTGGTTTTCCACTACCC
JUN	F	GGAGACAAGTGGCAGAGTCC
JUN	R	CCAAGTTCAACAACCGGTGC
RHOB	F	AAGCGAACTTTGTGCCTGTC
RHOB	R	ACAGCGTACAAGTGTGGTCA
TNFA	F	GAGGCCAAGCCCTGGTATG
TNFA	R	CGGGCCGATTGATCTCAGC
GAPDH	F	TCGGAGTCAACGGATTTGGT
GAPDH	R	TTCCCGTTCTCAGCCTTGAC
CCNE2	F	TAGCTGGTCTGGCGAGGTTTT
CCNE2	R	GGATTATCTGGGCTTCTTGGGG
AR	F	TGCTGTACAGGAGCCGAAGG
AR	R	CCAGGCACTTTCCTTGCTTC
NSE	F	TGCACAGGCCAGATCAAGAC
NSE	R	CCAGGCAAGCAGAGGAATCA

**ChIP-seq primers.**		
Gene name/ChIP	Forward (F) /Reverse (F	Sequence (5'-3')
GNB1L	F	CGGCCACTCTGGGAGTGT
GNB1L	R	CCAGGACGGAGTTTTTTCCCC
EDNRA	F	GCCCACATCTTTAGCTCCCAAAG
EDNRA	R	CCAAAGGTTTGGCACAACATTATGT
SSTR1	F	GGAGGGTAGACCATTTGGGG
SSTR1	R	GCTTCCTTCTTACAGCCCGG
GRIN3A	F	GCCCATGGACCAATCTCATTCC
GRIN3A	R	CGGGGTAGGGATACAAGTGAGTA
GRM4	F	CACACGAAGGCCTCTCTTCTG
GRM4	R	CTCTGGGCTGTTTACCCGC

The oligonucleotides sequence of shRNAs.

shRNA name	Sequence (5'-3')
shRB1-1	CCGGGTGCGCTCTT GAGGTTGTAATCTC GAGATTACAACCT
shRB1-2	CAACACCCCACTT TGTATTGAGATTCT CGAGAATCTCAAT
shCDK4-1	ACACGATCTCTGTT CTCGAGATGTACTC GAGTACATCTCGA
shCDK4-2	GGCCAGTCATCTTT GAGGTGGCTTTACT CGAGTAAAGCCAC
shCDK4-3	CTCACGAACTGTTT CTGGACAAGGCAC TCGAGTGCCTTGTC
shCDK6-1	CAGATATGTCCTTT TTGGCTGCATATCT CGAGATATGCAGC
shCDK6-2	CAACACTCCAGAT TTCCTATCTTAACT CGAGTTAAGATAG
shCDK6-3	GAACATCTCATGTT GTAACAGATATCC TCGAGGATATCTGT
shE2F1-1	TACAAACTTCTCTT ACCTCACTGAATCT CGAGATTCAGTGA GGTCTCA
shE2F1-2	TTCGGAGAACTTCT CGAGAAGTTCTCC GAAGAGT

Primers for subclone

Name	Forward (F) /Reverse (F	Sequence (5'-3')	Vector
BD1 (BRD4)	F	CCGGAATTCCGGAGCACCAACCCCCCGC	pGEX4T-1
BD1 (BRD4)	R	CCGCTCGAGCGGTTCTTCTGTGGGTAGCT CATTTATTTTTTGCAAG	pGEX4T-1
BD2 (BRD4)	F	CCGGAATTCCGGAAGGACGTGCCCGACTC TCAG	pGEX4T-1
BD2 (BRD4)	R	CCGCTCGAGCGGGTCCGGCATCTTGGCAA AGCG	pGEX4T-1

Primers for mutagenesis

BD1(F83A)	F	GCCCAGCAGCCTGTGGATGCCGTCAAGCTG
BD1(F83A)	R	AGGCCATGCAAACTGGTGTTTCCATAGTGTCTTGAGC
BD1(V87A)	F	CGGATGCCGTCAAGCTGAACCTCCCTGATTACTATAAG
BD1(V87A)	R	CAGGCTGCTGGGCAGGCCATGCAAAC
BD1(F157A)	F	GCCTTGCAAAAAATAAATGAGCTACCCACAGAAGAATGACTCGAGGG
BD1(F157A)	R	GAGCTTTTCCAGAGCTTCTGCCATTAAGACTATGTCATCTCC
BD1(161A)	F	GCAAATGAGCTACCCACAGAAGAATGACTCGAGGGG
BD1(I161A)	R	TTTTTGCAAGGCGAGCTTTTCCAGAGCTTCTGC
RB (R661W)	F	TGGCTAAATACACTTTGTGAACGCCTTC
RB (R661W)	R	GAGATAGGCTAGCCGATACACTTTTTTATAAAACAG
BD1 (E151A)	F	CAGCTCTGGAAAAGCTCTTCTTGCAAAAAATAAATG
BD1 (E151A)	R	CTGCCATTAAGACTATGTCATCTCCAGGC
BD1 (E154A)	F	CGCTACCCACAGAAGAACCGCTCGAG
BD1 (E154A)	R	CATTTATTTTTTGCAAGAAGAGCTTTTCCAGAGCTGC
BD1 (E163A)	F	CAAAGCTCTTCTTGCAAAAAATAAATGCGCTACCCAC
BD1 (E163A)	R	CCAGAGCTGCTGCCATTAAGACTATGTCATCTC
BD1 (E167A/E16	F	CAGCACCGCTCGAGCGGCCGCATCGTGAC
BD1 (E167A/E16	R	CTGTGGGTAGCGCATTTATTTTTTGCAAGAAGAGCTTTGCCAGAGC
BD1 (E151R)	F	AGAGCTCTGGAAAAGCTCTTCTTGCAAAAAATAAATGAGC
BD1 (E151R)	R	TGCCATTAAGACTATGTCATCTCCAGGCTTG
BD1 (E154R)	F	AGGCTACCCACAGAAGAACCGCTCGAGC
BD1 (E154R)	R	ATTTATTTTTTGCAAGAAGAGCTTTTCCAGAGCTCTTGCC
BD1 (E163R)	F	AGAAAGCTCTTCTTGCAAAAAATAAATAGGCTACCCACAGAAG
BD1 (E163R)	R	CAGAGCTCTTGCCATTAAGACTATGTCATCTCCAG

BD1 (E167R/E16{truncated}	F	AGAAGACCGCTCGAGCGGCCGCATCGTGAC
BD1 (E167R/E16{truncated}	R	TGTGGGTAGCCTATTTATTTTTTGCAAGAAGAGCTTTCTCAGAGCTCTTGCC

**Sequence for sgRNAs**		
sgGNB1L-1	F	CACCGGGATGGATCGGTGGTCCTGT
sgGNB1L-1	R	AAACACAGGACCACCGATCCATCCC
sgGNB1L-2	F	CACCGCTCATGAACTCACCAATCCC
sgGNB1L-2	R	AAACGGGATTGGTGAGTTCATGAGC
