# Supplementary material for: Retinoblastoma protein as an intrinsic BRD4 inhibitor modulates small molecule BET inhibitor sensitivity in cancer
Source: Nat Commun. 2022 Oct 23;13:6311. doi: 10.1038/s41467-022-34024-y (PMC9588789; doi:10.1038/s41467-022-34024-y)
Supplement: Supplementary file 3 — Description of additional Supplementary File [file 41467_2022_34024_MOESM3_ESM.pdf]

Descriptions of Additional Supplementary Data files

Supplementary Data 1. BRD4 ChIP-seq quality metrics determined by Cistrome analysis.

Supplementary Data 2. The genome loci with increased BRD4 occupancy after RB knockdown.

Supplementary Data 3. The genome loci with down-regulated BRD4 occupancy after RB knockdown.

Supplementary Data 4. Sequence information of PCR primers, shRNAs and sgRNAs.
