# Supplementary material for: Retinoblastoma protein as an intrinsic BRD4 inhibitor modulates small molecule BET inhibitor sensitivity in cancer
Source: Nat Commun. 2022 Oct 23;13:6311. doi: 10.1038/s41467-022-34024-y (PMC9588789; doi:10.1038/s41467-022-34024-y)
Supplement: Supplementary file 8 — Reporting Summary [file 41467_2022_34024_MOESM8_ESM.pdf]

nature portfolio

Corresponding author(s): Drs. Haojie Huang and Lei shi

Last updated by author(s): Sep 1, 2022

Reporting Summary

Nature Portfolio wishes to improve the reproducibility of the work that we publish. This form provides structure for consistency and transparency in reporting. For further information on Nature Portfolio policies, see our Editorial Policies and the Editorial Policy Checklist.

Statistics

For all statistical analyses, confirm that the following items are present in the figure legend, table legend, main text, or Methods section.

n/a	Confirmed	
☐	☒	The exact sample size (*n*) for each experimental group/condition, given as a discrete number and unit of measurement
☒	☐	A statement on whether measurements were taken from distinct samples or whether the same sample was measured repeatedly
☐	☒	The statistical test(s) used AND whether they are one- or two-sided<br>*Only common tests should be described solely by name; describe more complex techniques in the Methods section.*
☒	☐	A description of all covariates tested
☒	☐	A description of any assumptions or corrections, such as tests of normality and adjustment for multiple comparisons
☐	☒	A full description of the statistical parameters including central tendency (e.g. means) or other basic estimates (e.g. regression coefficient) AND variation (e.g. standard deviation) or associated estimates of uncertainty (e.g. confidence intervals)
☐	☒	For null hypothesis testing, the test statistic (e.g. *F*, *t*, *r*) with confidence intervals, effect sizes, degrees of freedom and *P* value noted<br>*Give P values as exact values whenever suitable.*
☒	☐	For Bayesian analysis, information on the choice of priors and Markov chain Monte Carlo settings
☒	☐	For hierarchical and complex designs, identification of the appropriate level for tests and full reporting of outcomes
☐	☒	Estimates of effect sizes (e.g. Cohen's *d*, Pearson's *r*), indicating how they were calculated

Our web collection on statistics for biologists contains articles on many of the points above.

Software and code

Policy information about availability of computer code

Data collection	UCSC genome browser (https://genome.ucsc.edu) was used to access and visualize gene tracks;<br>Odyssey Fc Imager-LI-COR Imaging to acquire the data from western blot;<br>CFX Maestro Software 2.3 to acquire data from RT-qPCR or ChIP-qPCR;<br>Soft Max Pro V5 to measure the OD values from cell growth assays and IC50 assays.
Data analysis	GraphPad Prism v8, Adobe Photoshop 2021, Adobe illustrate 2022, MACS2 (version 2.1.1), DEseq2, Genomic Regions Enrichment of Annotations Tool (GREAT), DiffBind software, Enrichr Databse (https://maayanlab.cloud/Enrichr/), Cistrome database BRD4 ChIP-seq samples, TCGA database.

For manuscripts utilizing custom algorithms or software that are central to the research but not yet described in published literature, software must be made available to editors and reviewers. We strongly encourage code deposition in a community repository (e.g. GitHub). See the Nature Portfolio guidelines for submitting code & software for further information.

Data

Policy information about availability of data

All manuscripts must include a data availability statement. This statement should provide the following information, where applicable:

- Accession codes, unique identifiers, or web links for publicly available datasets
- A description of any restrictions on data availability
- For clinical datasets or third party data, please ensure that the statement adheres to our policy

The ChIP-seq data has been deposited into at the NCBI's GEO data repository with the accession code GSE191263. The Human reference genome (GRCh38/hg38) used is accessible through GenBank/RefSeq assembly accession numbers GCA_000001405.15/GCA_000001405.26.

Field-specific reporting

Please select the one below that is the best fit for your research. If you are not sure, read the appropriate sections before making your selection.

☒ Life sciences ☐ Behavioural & social sciences ☐ Ecological, evolutionary & environmental sciences

For a reference copy of the document with all sections, see nature.com/documents/nr-reporting-summary-flat.pdf

Life sciences study design

All studies must disclose on these points even when the disclosure is negative.

Sample size	The sample size was determined based on our previous publication (PMIDs: 34397171, 33419772, 30527665, 30057199). The sample size of patients was determined by TCGA database.
Data exclusions	No data were excluded.
Replication	For the RT-qPCR and ChIP-qPCR assays, three replicate was performed;<br>For ChIP-seq assay, two replicate was performed.<br>For IC50 in Figure 1, FigureS7j, and FigureS8 h,i was determined from three independent replicate.<br>For colony formation assay, results were shown from three independent replicate.<br>For IC50 in Figure 5, FigureS2, and FigureS7b,d,f,h was determined by five independent replicate.<br>For cell growth assay, data was shown from five replicate. For the in vivo assay, data was collected and analyzed by the indication in manuscript.<br>For western blot, two independent technical replicate was performed to ensure the reproducibility. All the results from replicate for each experiment were consistent.<br>Others were indicated in the figure legends.
Randomization	For the IC50 or cell growth assay, cells were randomly divided into the indicated groups for the shRNAs infection or drug treatment. For the in vivo experiment, mice were allocated randomly for acquiring drug treatment.
Blinding	For the in vitro experiments, investigators involved in the group organization, treatment, sample collection were not blinded, while the investigators collecting data and generating output were blinded to all groups. For the in vivo experiments, investigators who were designing the organization of the groups and performing the treatment were not blinded to the groups and treatment, while other investigators collecting data such as tumor volume and tumor weight were blinded.

Reporting for specific materials, systems and methods

We require information from authors about some types of materials, experimental systems and methods used in many studies. Here, indicate whether each material, system or method listed is relevant to your study. If you are not sure if a list item applies to your research, read the appropriate section before selecting a response.

Materials & experimental systems

n/a	Involved in the study	
☐	☒	Antibodies
☐	☒	Eukaryotic cell lines
☒	☐	Palaeontology and archaeology
☐	☒	Animals and other organisms
☐	☒	Human research participants
☒	☐	Clinical data
☒	☐	Dual use research of concern

Methods

n/a	Involved in the study	
☐	☒	ChIP-seq
☒	☐	Flow cytometry
☒	☐	MRI-based neuroimaging

Antibodies

Antibodies used	The antibodies were indicated with their catalog numbers and dilution ratio.<br>Anti-RB (554136, BD bioscience), 1:1000 in dilution,<br>p107/RBL1 (SC-318, Santa Cruz Biotechnology), 1:500 in dilution,<br>p130/RBL2 (SC-317, Santa Cruz Biotechnology),1:500 in dilution,<br>ERK2 (D2) (SC-1647, Santa Cruz Biotechnology), 1:2000 in dilution,<br>HA (901515, Biolegend), 1:1000 in dilution,<br>FLAG (M2) (F-3165, Sigma), 1:1000 in dilution,<br>V5 (SC-81594, Santa Cruz Biotechnology), 1:1000 in dilution,<br>p50 (13586, Cell signaling technology), 1:1000 in dilution,<br>CDK4 (SC-601, Santa Cruz Biotechnology), 1:800 in dilution,

CDK6 (SC-177, Santa Cruz Biotechnology), 1:800 in dilution,
Histone H3 (dilution 1:3000, 9715, CST), 1:1000 in dilution,
BRD2 (ab139690, Abcam), 1:1000 in dilution,
BRD3 (A302-368A, Bethyl Lab), 1:1000 in dilution,
BRD4 (A301-985A100, Bethyl Lab), 1:1000 in dilution,
BRD4 (ab128874, Abcam), 1:1000 in dilution,
GNB1L (HPA034627, Sigma), 1:1000 in dilution,
Cleaved PARP (5625, Cell signaling technology), 1:1000 in dilution,
Cleaved Caspase-3 (Asp175) (9661, Cell signaling technology), 1:1000 in dilution,
Anti-mouse secondary antibody (115-035-003, Jackson Immunoresearch), 1:10000 in dilution,
Anti-mouse secondary antibody, light chain specific (115-035-174, Jackson Immunoresearch), 1:5000 in dilution,
Anti-rabbit secondary antibody (111-035-144, Jackson Immunoresearch). anti-Ki67 (ab15580, Abcam). 1:10000 in dilution,

Validation

All the antibodies were validated in the application of WB or IHC or ChIP/ChIP-seq according to their manufactures' instruction.
The commercial information for the antibodies are as following:
Anti-RB (554136, BD bioscience): https://www.bdbiosciences.com/en-au/products/reagents/flow-cytometry-reagents/research-reagents/single-color-antibodies-ruo/purified-mouse-anti-human-retinoblastoma-protein.554136
p107/RBL1 (SC-318, Santa Cruz Biotechnology): https://datasheets.scbt.com/sc-318.pdf
p130/RBL2 (SC-317, Santa Cruz Biotechnology): https://datasheets.scbt.com/sc-317.pdf
ERK2 (D2) (SC-1647, Santa Cruz Biotechnology): https://datasheets.scbt.com/sc-1647.pdf
HA (901515, Biolegend): https://www.biolegend.com/en-us/global-elements/pdf-popup/anti-ha-11-epitope-tag-antibody-11071
FLAG (M2) (F-3165, Sigma): https://www.sigmaaldrich.com/US/en/product/sigma/f3165
V5 (SC-81594, Santa Cruz Biotechnology): https://www.scbt.com/p/v5-probe-antibody-e10
p50 (13586, Cell signaling technology): https://www.cellsignal.com/products/primary-antibodies/nf-kb1-p105-p50-d4p4d-rabbit-mab/13586
CDK4 (SC-601, Santa Cruz Biotechnology): https://datasheets.scbt.com/sc-601.pdf
CDK6 (SC-177, Santa Cruz Biotechnology): https://www.scbt.com/p/cdk6-antibody-b-10
Histone H3 (dilution 1:3000, 9715, CST): https://www.cellsignal.com/products/primary-antibodies/histone-h3-antibody/9715
BRD2 (ab139690, Abcam): https://www.abcam.com/brd2-antibody-epr7642-chip-grade-ab139690.html
BRD3 (A302-368A, Bethyl Lab): https://www.fortislife.com/products/primary-antibodies/rabbit-anti-brd3-antibody/BETHYL-A302-368
BRD4 (A301-985A100, Bethyl Lab): https://www.fortislife.com/products/primary-antibodies/brd4-antibody/BETHYL-A301-985
BRD4 (ab128874, Abcam): https://www.abcam.com/brd4-antibody-epr51502-ab128874.html
GNB1L (HPA034627, Sigma): https://www.sigmaaldrich.com/US/en/product/sigma/hpa034627
Cleaved PARP (5625, Cell signaling technology): https://www.cellsignal.com/products/primary-antibodies/cleaved-parp-asp214-d64e10-xp-rabbit-mab/5625
Cleaved Caspase-3 (Asp175) (9661, Cell signaling technology): https://www.cellsignal.com/products/primary-antibodies/cleaved-caspase-3-asp175-antibody/9661
anti-Ki67 (ab15580, Abcam): https://www.abcam.com/ki67-antibody-ab15580.html
Peroxidase AffiniPure Goat Anti-Mouse IgG, light chain specific: https://www.jacksonimmuno.com/catalog/products/115-035-174
Peroxidase AffiniPure Goat Anti-Mouse IgG (H+L): https://www.jacksonimmuno.com/catalog/products/115-035-003
Peroxidase AffiniPure Goat Anti-Rabbit IgG (H+L): https://www.jacksonimmuno.com/catalog/products/111-035-144

Eukaryotic cell lines

Policy information about cell lines

Cell line source(s)	PC-3, DU-145, LNCaP and 293T cells were purchased from American Type Culture Collection (ATCC). C4-2 cells were purchased from Uro Corporation.
Authentication	Cell types were authenticated by morphology and/or western blot.
Mycoplasma contamination	All the cell lines were tested negative for mycoplasma contamination during the study.
Commonly misidentified lines (See ICLAC register)	No misidentified lines reported.

Animals and other organisms

Policy information about studies involving animals; ARRIVE guidelines recommended for reporting animal research

Laboratory animals	Six-week old male SCID mice (generated in house) were used. All mice were housed in 22°C, 55% humidity on average with a 12-h light/12-h dark cycle and access to food and water.
Wild animals	No wild animals used in this study.
Field-collected samples	No field-collected samples were involved in this study.
Ethics oversight	Mice experiment was approved by the Institutional Animal Care and Use Committee (IACUC) at the Mayo Clinic.

Note that full information on the approval of the study protocol must also be provided in the manuscript.

Human research participants

Policy information about studies involving human research participants

Population characteristics	Metastatic PCa specimens were obtained from patients undergoing standard-of-care biopsies at Mayo Clinic (Rochester, MN).
Recruitment	Samples were collected from the Mayo Clinic (Rochester, MN) and annotated for major clinic-pathologic variables through review of pathology reports and clinical records by trained personnel.
Ethics oversight	The study was carried out with the approval of the institutional review board (IRB) of Mayo Clinic.

Note that full information on the approval of the study protocol must also be provided in the manuscript.

ChIP-seq

Data deposition

- [x] Confirm that both raw and final processed data have been deposited in a public database such as GEO.
- [x] Confirm that you have deposited or provided access to graph files (e.g. BED files) for the called peaks.

Data access links
May remain private before publication.

The ChIP-seq data is available at https://www.ncbi.nlm.nih.gov/geo/query/acc.cgi?acc=GSE191263.
The token for the link is included in the cover letter.

Files in database submission

Accession Title Release date Status Supplemen-
tary files

GSE191263 RB-N phosphorylation promotes BRD4-mediated CREB signaling hyperactivation and BET inhibitor resistance Dec 16, 2022 approved None

GSM5742621	shcon-Rep1-BRD4	Dec 16, 2022	approved	BW
GSM5742622	shcon-Rep2-BRD4	Dec 16, 2022	approved	BW
GSM5742623	shcon-Rep1-H4K5Ac	Dec 16, 2022	approved	BW
GSM5742624	shcon-Rep2-H4K5Ac	Dec 16, 2022	approved	BW
GSM5742625	shRB-Rep1-BRD4	Dec 16, 2022	approved	BW
GSM5742626	shRB-Rep2-BRD4	Dec 16, 2022	approved	BW
GSM5742627	shRB-Rep1-H4K5Ac	Dec 16, 2022	approved	BW
GSM5742628	shRB-Rep2-H4K5Ac	Dec 16, 2022	approved	BW

Genome browser session
(e.g. UCSC)

No longer applicable.

Methodology

Replicates

2 biological replicated

Sequencing depth

GSM5742621	shcon-Rep1-BRD4	83257539 unique mapped reads in total of 85910584 reads.
GSM5742622	shcon-Rep2-BRD4	76484645 unique mapped reads in total of 85221052 reads.
GSM5742623	shcon-Rep1-H4K5Ac	98178804 unique mapped reads in total of 100160370 reads.
GSM5742624	shcon-Rep2-H4K5Ac	75528761 unique mapped reads in total of 77374788 reads.
GSM5742625	shRB-Rep1-BRD4	87911948 unique mapped reads in total of 90538126 reads.
GSM5742626	shRB-Rep2-BRD4	87190354 unique mapped reads in total of 89677324 reads.
GSM5742627	shRB-Rep1-H4K5Ac	86080079 unique mapped reads in total of 88423448 reads.
GSM5742628	shRB-Rep2-H4K5Ac	100047395 unique mapped reads in total of 102169718 reads.

All the data is Paired-end with 51 bp of each read.

Antibodies

BRD4 (A301-985A100, Bethyl Lab), BRD4 (ab128874, Abcam).

Peak calling parameters

The raw reads were subjected to the human reference genome (GRCh37/hg38) using bowtie2 (version 2.2.9). MACS2 (version 2.1.1) was run to perform the peak calling with a p value threshold of 1 × 10-5.

Data quality

There are 17909 and 18559 filtered peaks for shcon-BRD4,
there are 51677 and 47811 filtered peaks for shcon-H4K5,
there are 14445 and 15285 filtered peaks for shRB-BRD4,
there are 60446 and 56337 filtered peaks for shRB-H4K5.

Software

Bowtie2 (version 2.2.9) for the row reads generation,
MACS2 (version 2.1.1) was run to perform the peak calling,
Genomic Regions Enrichment of Annotations Tool (GREAT) for the assignment of peaks to potential target genes.
